# Vitamin D Deficiency and the Clinical Outcomes of Calcimimetic Therapy in Dialysis Patients: A Population-Based Study

**DOI:** 10.3390/nu17091536

**Published:** 2025-04-30

**Authors:** Kuo-Cheng Lu, Joshua Wang, Cai-Mei Zheng, Kuo-Wang Tsai, Yi-Chou Hou, Chien-Lin Lu

**Affiliations:** 1 Division of Nephrology, Department of Medicine, Taipei Tzu Chi Hospital, Buddhist Tzu Chi Medical Foundation, New Taipei City 23142, Taiwan; tch33730@tzuchi.com.tw; 2 Department of Research, Taipei Tzu Chi Hospital, Buddhist Tzu Chi Medical Foundation, New Taipei City 23142, Taiwan; j3.reilly@qut.edu.au; 3 School of Biomedical Sciences, Queensland University of Technology, Brisbane 4001, Australia; 4 Division of Nephrology, Department of Internal Medicine, Shuang Ho Hospital, School of Medicine, College of Medicine, Taipei Medical University, New Taipei City 11031, Taiwan; 11044@s.tmu.edu.tw; 5 TMU Research Centre of Urology and Kidney, Taipei Medical University, New Taipei City 11031, Taiwan; 6 Division of Nephrology, Department of Internal Medicine, Cardinal-Tien Hospital, School of Medicine, College of Medicine, Fu Jen Catholic University, New Taipei City 24205, Taiwan; tch33225@tzuchi.com.tw; 7 School of Medicine, College of Medicine, Fu Jen Catholic University, New Taipei City 24205, Taiwan; 127097@mail.fju.edu.tw; 8 Division of Nephrology, Department of Internal Medicine, Fu Jen Catholic University Hospital, Fu Jen Catholic University, New Taipei City 24352, Taiwan

**Keywords:** calcimimetic therapy, cardiovascular events, fracture risk, secondary hyperparathyroidism, vitamin D deficiency

## Abstract

Background: Vitamin D deficiency (VDD) is prevalent in patients with secondary hyperparathyroidism (SHPT) undergoing dialysis and may attenuate the efficacy of calcimimetic therapy, which is designed to reduce parathyroid hormone (PTH) levels and improve clinical outcomes. This study aimed to investigate the impact of vitamin D status on all-cause mortality, major adverse cardiovascular events (MACEs), fractures, and hypocalcemia in dialysis patients receiving calcimimetics. Methods: This retrospective cohort study utilized the TriNetX database to identify dialysis patients treated with calcimimetics between 2010 and 2024. Patients were classified into VDD (<20 ng/mL) and vitamin D-adequate (VDA, ≥30 ng/mL) groups. Propensity score matching (1:1) was performed on 95 covariates to minimize confounding. Outcomes, including all-cause mortality, MACEs, fractures, hypocalcemia, and PTH suppression (≤300 pg/mL), were compared between groups over a 3-year follow-up. Multiple comparisons were adjusted using the Bonferroni–Holm correction. Results: All-cause mortality was significantly higher in the VDD group (25.4%) compared to the VDA group (20.9%), with an adjusted odds ratio (OR) of 1.29 (95% CI: 1.10–1.51, *p* = 0.002, corrected α = 0.007). While initial analyses suggested associations between VDD and the increased risks of MACEs, fractures, and hypocalcemia, these results did not remain significant after correction. Subgroup analysis indicated that comorbidities, such as obesity, dyslipidemia, and depression, amplified these risks in the VDD group. No significant differences were observed for PTH suppression (≤300 pg/mL) between groups. Conclusions: VDD is independently associated with increased all-cause mortality in dialysis patients with SHPT, even after multiple comparison adjustments. While risks for MACEs, fractures, and hypocalcemia showed non-significant trends, their observed patterns suggest potential clinical relevance. Optimizing vitamin D status may enhance clinical outcomes in this high-risk population, warranting further investigation through randomized controlled trials.

## 1. Introduction

Cardiovascular disease is the primary cause of death in patients with chronic kidney disease (CKD), with mortality rates that are over ten times higher than those in the general population [1]. This heightened risk is predominantly attributed to secondary hyperparathyroidism (SHPT), a frequent complication in CKD characterized by disruptions in the calcium–phosphorus metabolism and the elevated secretion of parathyroid hormone (PTH). PTH receptors are constitutively expressed in cardiovascular cells such as cardiomyocytes, endothelial cells, and smooth muscle cells [2]. Elevated PTH levels (>600 pg/mL) markedly promote vascular calcification, myocardial hypertrophy, and endothelial dysfunction, which collectively contribute to adverse cardiovascular outcomes and higher mortality rates in CKD patients [3,4].

SHPT management frequently involves calcimimetic agents, such as cinacalcet and etelcalcetide, which target calcium-sensing receptors (CaSRs) to reduce PTH levels and stabilize calcium–phosphorus metabolism. The EVOLVE trial examined the cardiovascular effects of cinacalcet in dialysis patients with SHPT and demonstrated a significant reduction in overall mortality after adjusting for baseline demographic differences [5,6]. Furthermore, the pooled analyses of observational data and randomized trials revealed a 9% reduction in all-cause mortality risk with cinacalcet therapy [7]. Etelcalcetide has exhibited superior efficacy in patients inadequately managed with cinacalcet, significantly reducing extremely high PTH levels and improving cardiovascular outcomes, including left ventricular hypertrophy [8]. However, despite these benefits, calcimimetic efficacy can be modulated by additional factors, such as vitamin D deficiency (VDD).

VDD is widespread among CKD patients and worsens SHPT by impairing intestinal calcium absorption, promoting vascular calcification, and exacerbating systemic inflammation [9]. Meta-analyses have shown that each 10 ng/mL increase in serum 25(OH)D is associated with significant reductions in cardiovascular and all-cause mortality risks [10]. Correcting VDD could therefore enhance the therapeutic benefits of calcimimetic agents in CKD patients.

This study investigated the relationships among VDD, PTH control, and clinical outcomes during calcimimetic therapy in a large multi-institutional cohort of dialysis patients with SHPT. By analyzing the association of vitamin D levels with cardiovascular events, mortality, and bone health, this study sought to establish potential evidence-based strategies to improve long-term outcomes in this high-risk population.

## 2. Methods

### 2.1. Study Design

This retrospective cohort study was performed using the TriNetX database and complied with the Strengthening the Reporting of Observational Studies in Epidemiology (STROBE) guidelines (Supplementary Data S1). TriNetX is a global network of electronic health records from healthcare organizations that permits the querying of deidentified patient data [11]. The de-identification of patient data had been assessed as being compliant with Section §164.514(b)(1) of the HIPAA Privacy Rule. This study was approved by the Taipei Tzu Chi Hospital Institutional Review Board (approval number: 13-IRB133). The Global Collaborative Network, constituting 52 healthcare organizations, was queried for ICD-10 codes that matched the inclusion and exclusion criteria of the study (Supplementary Data S2). All data were collected on 8 December 2024.

### 2.2. Study Cohorts

The study evaluated 7043 patients from the TriNetX database who underwent maintenance dialysis (ICD-10: N18.6) between 2010 and 2024 and had received calcimimetic therapy (cinacalcet or etelcalcetide).

Patients with serum or plasma 25(OH)D levels < 20 ng/mL within 6 months prior to or 5 years after the initiation of calcimimetic therapy were categorized into the VDD cohort. To ensure assay consistency, only 25(OH)D values coded under the standardized LOINC code 35365-6 were included, corresponding to total 25-hydroxyvitamin D testing in mass concentration units [12]. TriNetX’s built-in data quality algorithms further exclude implausible or extreme laboratory values, improving reliability and minimizing inter-laboratory variability [11]. Individuals were excluded from the VDD cohort if any of their 25(OH)D3 measurements exceeded 20 ng/mL. Similarly, patients were included in the vitamin D-adequate (VDA) cohort if their 25(OH)D3 levels were ≥30 ng/mL within the same period, with exclusion applied for any measurements below 30 ng/mL.

Further exclusion criteria included patients aged <20 years or those with a documented history of primary hyperparathyroidism or benign/malignant parathyroid neoplasms, resulting in 2714 patients in the VDD group and 2410 patients in the VDA group. To reduce bias and achieve covariate balance, 1:1 propensity score matching was conducted, adjusting for factors such as age, sex, race, comorbidities, medication usage, and relevant laboratory parameters 3 years prior to calcimimetic use. The 1:1 propensity score matching of 95 variables between the VDD and VDA groups was performed. The greedy nearest neighbor algorithm was used with a caliper of 0.1 pooled standard deviations. A summary of these variables before and after propensity score matching is shown in Table 1.

**Figure 1:**
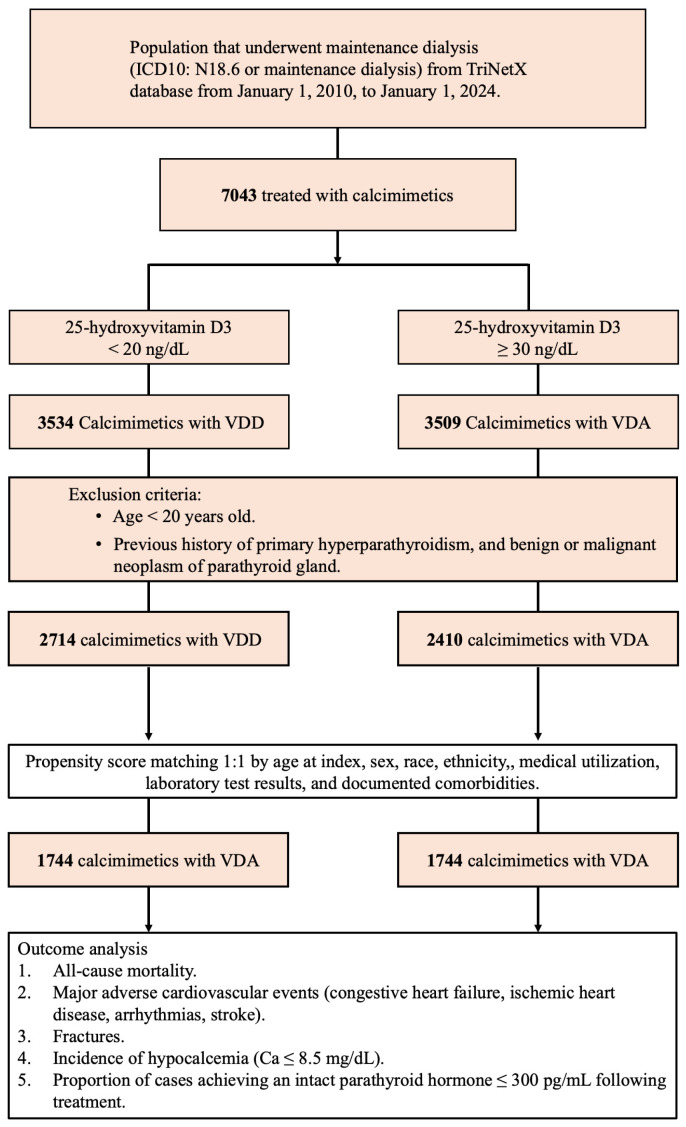
Flowchart of patient selection and enrollment in this study.

The final analysis included 1744 matched patients in each group, enabling robust comparisons of clinical outcomes, including overall mortality, major adverse cardiovascular events (MACEs), fracture incidence, hypocalcemia rates, and PTH suppression (≤300 pg/mL), as shown in Figure 1.

### 2.3. Data Analysis

The propensity-matched cohorts were compared to assess their clinical outcomes three years after their first incidence of concurrent dialysis and calcimimetic treatment. Aside from all-cause mortality, the incidence of various comorbidities, including heart failure, MACEs, and fractures, was also assessed. Endocrine outcomes were also compared between the two cohorts, including the incidence of PTH ≤ 300 and hypocalcemia. The risk of each outcome was calculated for each cohort and used to determine the risk ratio of VDD. Independent two-sample *t*-tests were performed to determine if the outcome risk was significantly different between the VDD and VDA groups. Survival probabilities were also calculated using the Kaplan–Meier method, with *p*-values calculated using Cox proportional hazards regression models. To account for multiple comparisons, the Bonferroni–Holm correction was applied [13]. A total of seven tests were performed; the significance level for each test was adjusted accordingly. Therefore, an adjusted α of 0.00714 was used to determine statistical significance for the smallest *p*-value. For subsequent *p*-values, thresholds were adjusted iteratively based on the Bonferroni–Holm procedure. Results were reported with both raw *p*-values and corrected *p*-values to enhance transparency.

## 3. Results

### 3.1. Geographic Distribution of Patients

To explore the potential geographic confounding, especially related to seasonal variation in vitamin D levels, we assessed the regional distribution of patients in the VDD group using TriNetX’s network-specific filters. The majority of included patients were from the United States (n = 2192), followed by a small number from Europe and the Middle East (n = 106). No patients were identified from Asia–Pacific or Latin America regions. This finding suggests that our cohort primarily reflects US-based clinical data, thereby minimizing the impact of seasonal variations in sunlight exposure and vitamin D synthesis (Table 2).

### 3.2. All-Cause Mortality

**Figure 2:**
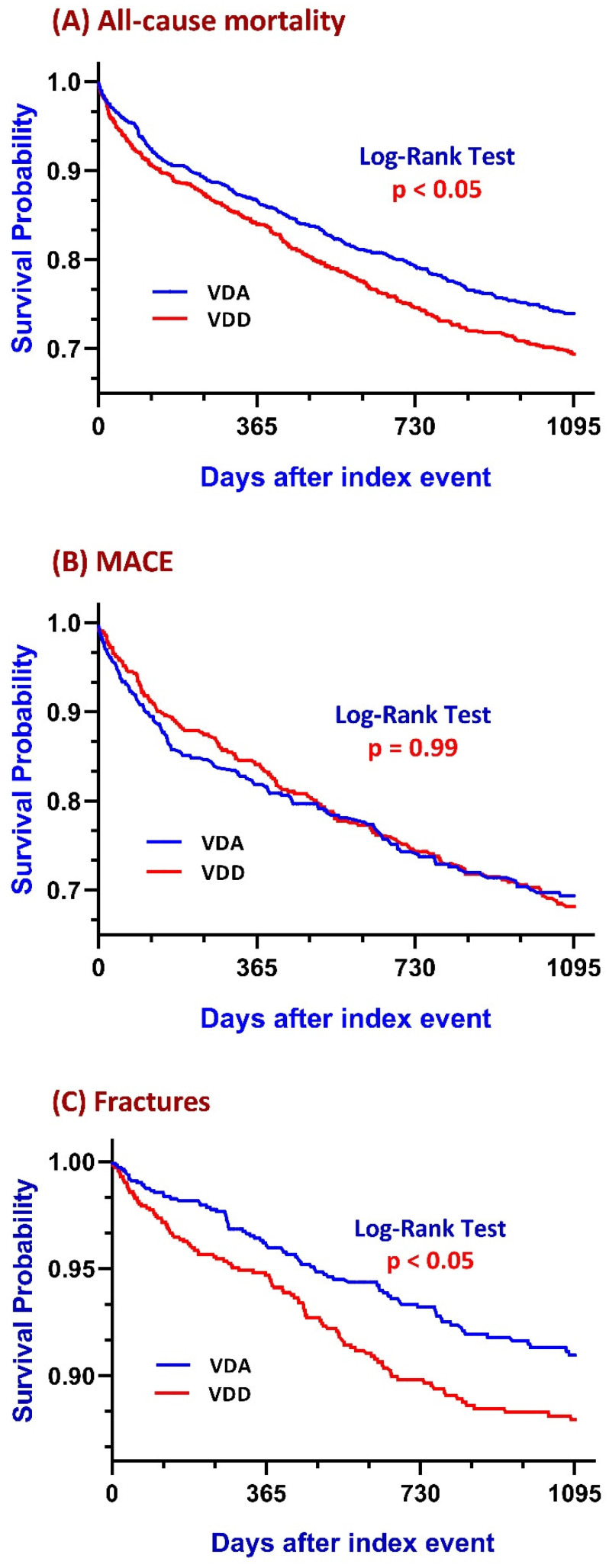
Kaplan–Meier survival curves for all-cause mortality, major adverse cardiovascular events, and fracture risk. This figure presents Kaplan–Meier survival curves comparing outcomes between the vitamin D-deficient (VDD) and vitamin D-adequate (VDA) groups: panel (**A**) illustrates all-cause mortality, where the VDD group demonstrated a significantly higher risk compared to the VDA group (HR = 1.23, 95% CI: 1.07–1.41; log-rank *p* = 0.004); panel (**B**) shows survival analysis for MACEs, with no statistically significant difference observed between the VDD and VDA groups (log-rank *p* = 0.99); and panel (**C**) depicts fracture risk, indicating a significantly increased risk in the VDD group compared to the VDA group (log-rank *p* < 0.05).

Patients in the VDD group exhibited a higher mortality risk of 25.4% compared to 20.9% in the VDA group. The risk difference was 0.05 (95% confidence interval (CI): 0.02–0.07), with an odds ratio (OR) of 1.29 (95% CI: 1.10–1.50). The original *p*-value was 0.002, which remained statistically significant after Bonferroni–Holm correction (corrected α = 0.007). Kaplan–Meier survival analysis revealed a significantly lower survival probability in the VDD group at the end of the study period (69.18%) compared to that of the VDA group (73.86%). The log-rank test confirmed a significant difference between the groups (log-rank *p* < 0.05) (Figure 2A). The hazard ratio (HR) was 1.23 (95% CI: 1.07–1.41).

**Figure 3:**
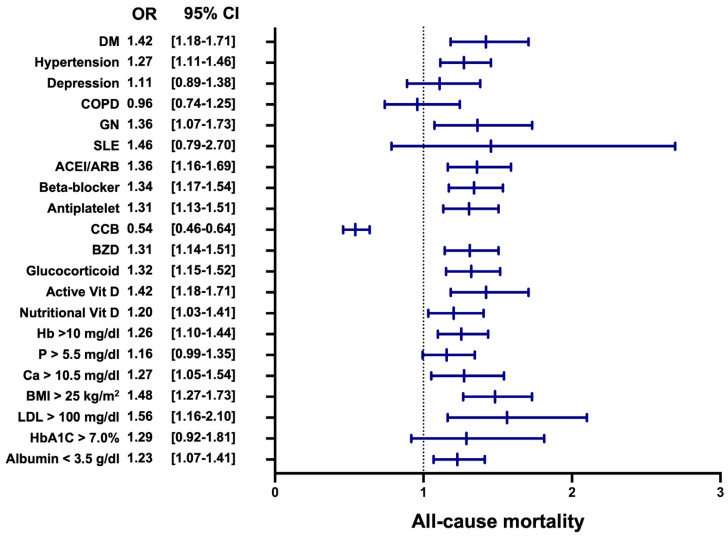
Forest plot of all-cause mortality in subgroup analysis. This figure shows the odds ratios for all-cause mortality across different subgroups. The subgroup analysis highlights various factors significantly associated with all-cause mortality. Comorbidities such as diabetes mellitus, hypertension, and glomerulonephritis were linked to higher mortality risk. Medication usage, including ACE inhibitors/ARBs, beta-blockers, antiplatelet agents, benzodiazepines, and glucocorticoids, showed increased risks. Laboratory and nutritional markers like low hemoglobin, hypoalbuminemia, hypercalcemia, obesity, and elevated LDL cholesterol also contributed to heightened mortality. LDL: low-density lipoprotein; BMI: body mass index; Ca: calcium; P: phosphate; Hb: hemoglobin; Vit D: vitamin D; BZD: benzodiazepines; CCB: calcium channel blocker; ACEI: angiotensin-converting enzyme inhibitor; ARB: angiotensin II receptor blocker; SLE: systemic lupus erythematosus; GN: glomerulonephritis; COPD: chronic obstructive pulmonary disease; DM: diabetes mellitus.

The subgroup analysis of mortality demonstrated that several factors significantly influenced the mortality risk, with most results indicating worse outcomes in the VDA group (Figure 3). Among comorbid conditions, diabetes mellitus (OR: 1.42, 95% CI: 1.18–1.70, *p* < 0.01), hypertension (OR: 1.27, 95% CI: 1.11–1.46, *p* < 0.01), and glomerulonephritis (OR: 1.36, 95% CI: 1.07–1.73, *p* = 0.01) were associated with significantly increased mortality. Medication usage also influenced outcomes, with higher mortality risks observed in those using angiotensin-converting enzyme inhibitor (ACEI)/angiotensin II receptor blockers (ARBs) (OR: 1.36, 95% CI: 1.16–1.59, *p* < 0.01), beta-blockers (OR: 1.34, 95% CI: 1.17–1.54, *p* < 0.01), antiplatelet agents (OR: 1.31, 95% CI: 1.13–1.51, *p* < 0.01), benzodiazepines (OR: 1.31, 95% CI: 1.14–1.51, *p* < 0.01), and glucocorticoids (OR: 1.32, 95% CI: 1.15–1.52, *p* < 0.01). Conversely, calcium channel blockers demonstrated a protective effect, with an OR of 0.54 (95% CI: 0.46–0.64, *p* < 0.01) favoring the VDD group.

Nutritional and laboratory markers further highlighted disparities. Low nutritional vitamin D levels were associated with higher mortality (OR: 1.20, 95% CI: 1.03–1.41, *p* = 0.02), along with low hemoglobin (Hb > 10 g/dL, OR: 1.26, 95% CI: 1.10–1.44, *p* < 0.01), hypercalcemia (Ca > 10.5 mg/dL, OR: 1.27, 95% CI: 1.05–1.54, *p* = 0.01), and hypoalbuminemia (albumin < 3.5 g/dL, OR: 1.23, 95% CI: 1.07–1.41, *p* < 0.01). Additionally, obesity (body mass index (BMI) > 25 kg/m^2^, OR: 1.48, 95% CI: 1.27–1.73, *p* < 0.001) and elevated low-density lipoprotein (LDL) cholesterol (>100 mg/dL, OR: 1.56, 95% CI: 1.16–2.10, *p* < 0.01) were associated with increased mortality risk. These findings emphasize the multifactorial nature of mortality risk, with most subgroup analysis results indicating worse outcomes in the VDD group, highlighting the potential protective role of adequate vitamin D levels.

### 3.3. Major Adverse Cardiovascular Events

The risk of MACEs was modestly higher in the VDD group (24.6%) compared to the VDA group (22.8%), with an insignificant OR of 1.10 (95% CI: 0.87–1.40). The original *p*-value was 0.43; it remained non-significant after Bonferroni–Holm correction (corrected α = 0.03). Kaplan–Meier analysis showed survival probabilities of 69.32% in the VDD group and 71.10% in the VDA group. However, the log-rank test indicated no statistical significance (log-rank *p* = 0.99), with an HR of 1.07 (95% CI: 0.87–1.32) (Figure 2B).

**Figure 4:**
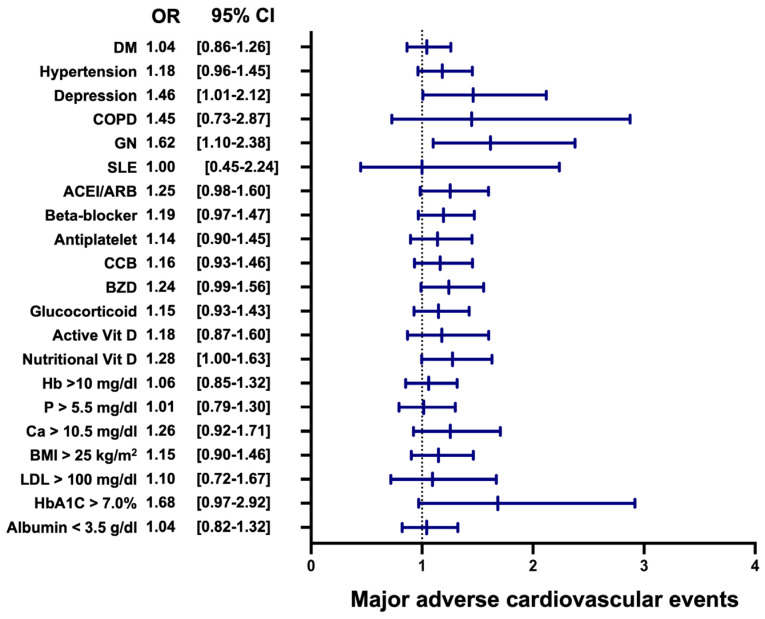
Forest plot of major adverse cardiovascular events in subgroup analysis. This figure illustrates the odds ratios for MACEs across various subgroups, highlighting key risk factors. Subgroups include LDL, BMI, hypertension, and glomerulonephritis, among others. LDL: low-density lipoprotein; BMI: body mass index; Ca: calcium; P: phosphate; Hb: hemoglobin; Vit D: vitamin D; BZD: benzodiazepines; CCB: calcium channel blocker; ACEI: angiotensin-converting enzyme inhibitor; ARB: angiotensin II receptor blocker; SLE: systemic lupus erythematosus; GN: glomerulonephritis; COPD: chronic obstructive pulmonary disease; DM: diabetes mellitus.

The subgroup analysis for MACEs highlighted significant differences between the VDD group and the VDA group (Figure 4). Patients with depression showed a significantly higher risk of MACEs in the VDD group, with an OR of 1.46 (95% CI: 1.01–2.12, *p* < 0.05). Similarly, those with glomerulonephritis (GN) demonstrated an increased risk of MACEs in the VDD group, with an OR of 1.62 (95% CI: 1.10–2.38, *p* = 0.02). Although other subgroups, such as patients with hypertension, those on ACE inhibitors/ARBs or beta-blockers, or those with low nutritional vitamin D, showed non-significant trends (*p* > 0.05), the OR was generally higher in the VDD group. The pooled analysis confirmed an overall increased risk of MACEs in the VDD group, with a pooled OR of 1.17 (95% CI: 1.10–1.24, *p* < 0.01). These findings suggest that VDD is associated with a heightened risk of MACEs among patients with COPD and GN, underscoring the potential cardiovascular benefits of maintaining adequate vitamin D levels.

### 3.4. Fracture

The fracture incidence was significantly greater in the VDD group (7.7%) compared to the VDA group (5.8%), with an OR of 1.34 (95% CI: 1.01–1.79). The original *p*-value was 0.044; after Bonferroni–Holm correction, it did not remain significant (corrected α = 0.01). Kaplan–Meier analysis highlighted survival probabilities of 89.10% and 91.55% for the VDD group and the VDA group, respectively, at the study’s end. The log-rank test demonstrated a significant difference (log-rank *p* < 0.05), with an HR of 1.37 (95% CI: 1.04–1.81) (Figure 2C).

**Figure 5:**
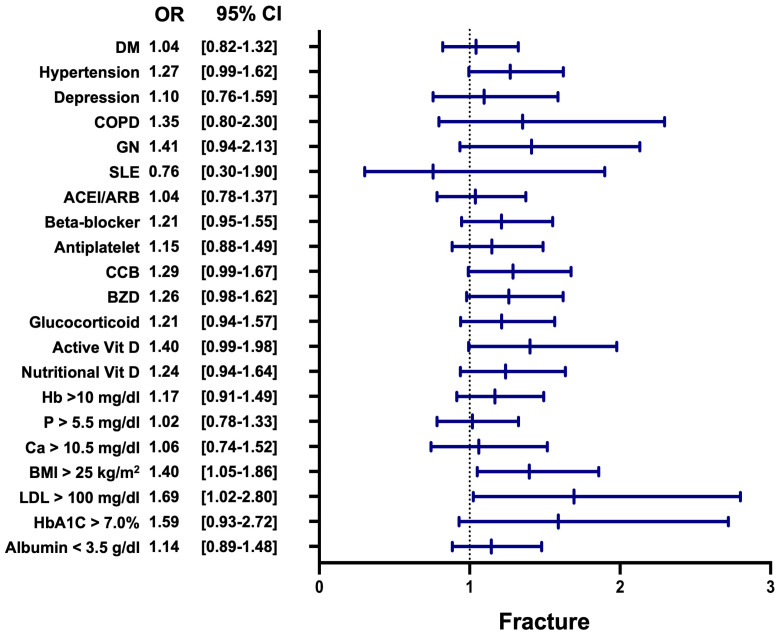
Forest plot of fracture risk in subgroup analysis. This figure presents the odds ratios for fracture incidence across subgroups. Key variables include BMI and LDL, illustrating significant contributors to fracture risks in the analyzed cohorts. LDL: low-density lipoprotein; BMI: body mass index; Ca: calcium; P: phosphate; Hb: hemoglobin; Vit D: vitamin D; BZD: benzodiazepines; CCB: calcium channel blocker; ACEI: angiotensin-converting enzyme inhibitor; ARB: angiotensin II receptor blocker; SLE: systemic lupus erythematosus; GN: glomerulonephritis; COPD: chronic obstructive pulmonary disease; DM: diabetes mellitus.

The subgroup analysis for fracture risk revealed that patients in the VDD group had a significantly higher risk of fractures compared to those in the VDA group (Figure 5). Among the subgroups, patients with a BMI > 25 kg/m^2^ demonstrated a significantly increased fracture risk, with an OR of 1.40 (95% CI: 1.05–1.86, *p* = 0.02). Similarly, elevated LDL cholesterol levels (>100 mg/dL) were associated with higher fracture risk in the VDD group, with an OR of 1.69 (95% CI: 1.02–2.80, *p* = 0.04). Although other subgroups, such as patients with hypertension, active vitamin D use, and hemoglobin A1c (HbA1c) > 7.0%, showed non-significant trends, most odds ratios favored reduced fracture risk in the VDA group.

### 3.5. Incidence of Hypocalcemia (Ca ≤ 8.5 mg/dL)

The occurrence of hypocalcemia was higher in the VDD group (45.0%) compared to the VDA group (36.6%), with an OR of 1.42 (95% CI: 1.08–1.87). The original *p*-value was 0.01; however, after the Bonferroni–Holm correction, the *p*-value did not retain significance (corrected α = 0.008). Kaplan–Meier analysis showed end-of-study survival probabilities of 44.50% in the VDD group and 53.33% in the VDA group. The log-rank test was significant (log-rank *p* = 0.01), with an HR of 1.32 (95% CI: 1.07–1.63).

### 3.6. Proportion of Cases with Intact Parathyroid Hormone (PTH ≤ 300 pg/mL) Following Treatment

The proportion of patients with PTH levels below 300 pg/mL was insignificantly lower in the VDD group (26.1%) than in the VDA group (26.7%), with an OR of 0.97 (95% CI: 0.76–1.23, *p* = 0.80). Kaplan–Meier analysis revealed survival probabilities of 65.04% in the VDD group and 66.74% in the VDA group; however, the log-rank test confirmed that this difference was not statistically significant (HR = 0.97, 95% CI: 0.79–1.19, log-rank *p* = 0.77).

## 4. Discussion

Our findings align with those of previous studies which show that VDD exacerbates risks associated with SHPT, including mortality, cardiovascular events, and fractures [14,15]. This study expands prior knowledge by providing detailed subgroup analyses and exploring how calcimimetic therapy interacts with vitamin D status. VDD aggravates systemic inflammation by increasing the production of pro-inflammatory cytokines [16], promoting vascular calcification through the transformation of vascular smooth muscle cells into osteoblast-like cells [17], and disrupting mineral metabolism by reducing calcium absorption and elevating phosphate and PTH levels [18]. It also contributes to RAAS overactivation, exacerbating hypertension and cardiorenal complications [19]. These mechanisms collectively increase morbidity and mortality, reducing the effectiveness of calcimimetic therapy.

The association between VDD and increased all-cause mortality in patients with SHPT is consistent with evidence linking vitamin D status to survival outcomes [14]. Importantly, the increased mortality risk observed in the VDD group remained significant after the Bonferroni–Holm correction for multiple testing, reinforcing the robustness of this finding. Mechanistically, VDD contributes to poor outcomes by impairing calcium absorption, disrupting bone remodeling, and altering systemic metabolic processes [20]. These changes exacerbate cardiovascular complications—the leading cause of death in dialysis patients. Recent studies suggest that vitamin D deficiency may also compromise host immune defense and increase vulnerability to severe infections, such as infective endocarditis, particularly in elderly or comorbid patients with prosthetic valves [21]. The inflammatory and immune-modulating roles of vitamin D have been linked to improved macrophage and dendritic cell function, which are frequently impaired in dialysis patients. Additionally, in patients with diabetes or obesity, vitamin D supplementation has shown modest improvements in insulin sensitivity and inflammatory control, suggesting a potential role in mitigating metabolic and vascular stress [22].

Our subgroup analysis further elucidated the clinical heterogeneity underlying mortality risk. Comorbidities, such as diabetes mellitus (OR: 1.42), hypertension (OR: 1.27), and glomerulonephritis (OR: 1.36), were independently associated with increased mortality, particularly in the context of VDD. These conditions likely interact with vitamin D deficiency through overlapping inflammatory and vascular mechanisms. For instance, VDD amplifies RAAS activation and oxidative stress in diabetic and hypertensive patients, while in glomerulonephritis, it may intensify immune dysregulation and endothelial injury [23,24].

Medication use also demonstrated a differential impact on mortality risk. ACE inhibitors/ARBs (OR: 1.36), beta-blockers (OR: 1.34), benzodiazepines (OR: 1.31), and glucocorticoids (OR: 1.32) were associated with increased mortality, potentially reflecting interactions with RAAS activity, altered pharmacodynamics under inflammatory stress, or enhanced susceptibility to adverse events in VDD states. In contrast, CCBs conferred a protective effect (OR: 0.54), likely due to their RAAS-independent hemodynamic benefits and potential to mitigate vascular calcification, even under VDD conditions [25].

Nutritional and laboratory parameters further underscored the complexity of mortality risk. Elevated LDL cholesterol (OR: 1.56) and BMI > 25 kg/m^2^ (OR: 1.48) reflect a pro-atherogenic and pro-inflammatory state, while hypoalbuminemia (OR: 1.23) and anemia (OR: 1.26) indicate the malnutrition–inflammation syndromes common in dialysis patients [26]. Hypercalcemia (OR: 1.27), often driven by the oversuppression of PTH or calcium-based phosphate binders, promotes arterial stiffness and calcific vasculopathy. Notably, low nutritional vitamin D levels (OR: 1.20) are independently correlated with mortality, supporting the notion that optimal vitamin D status may be protective beyond its skeletal benefits [27]. In the NECOSAD cohort, patients with 25(OH)D levels > 10 ng/mL had significantly lower short- and long-term mortality, particularly from cardiovascular causes [28]. A large German hemodialysis cohort reported that severe VDD (25(OH)D < 12.5 ng/mL) more than doubled the risk of all-cause mortality and was strongly associated with cardiac, infectious, and cancer mortality [29]. Furthermore, a meta-analysis of 18 cohorts (n > 14,000) showed that each 10 ng/mL increase in 25(OH)D was associated with a 22% reduction in all-cause mortality and a 29% reduction in cardiovascular mortality [30]. These findings reinforce our hypothesis that vitamin D adequacy contributes meaningfully to survival by mitigating cardiovascular, infectious, and metabolic risks in this vulnerable population. Together, these findings support the hypothesis that adequate vitamin D levels may mitigate mortality risk in dialysis patients by modulating inflammatory pathways, stabilizing vascular function, and enhancing the therapeutic responsiveness to calcimimetic agents. These observations warrant prospective validation and highlight the need to consider vitamin D optimization as part of a comprehensive risk stratification in patients with SHPT.

While the overall difference in MACE risk between VDD and VDA groups was insignificant, subgroup analyses revealed susceptible subpopulations, reinforcing the role of vitamin D in maintaining cardiovascular health, especially in dialysis patients. It is also possible that the lack of statistical significance reflects regression dilution bias, as our study utilized a single baseline 25(OH)D measurement rather than serial monitoring. A recent review emphasized that associations between low vitamin D status and major cardiovascular events may attenuate over longer follow-up periods due to intra-individual variability in 25(OH)D levels. This implies that the true cardiovascular risk associated with vitamin D deficiency might be underestimated when time-updated biomarker data are not available [31]. Additionally, seasonal factors may also influence cardiovascular outcomes. A large-scale ecological study across 19 countries demonstrated that cardiovascular mortality peaks in winter and is lowest in summer, particularly outside of tropical regions. This seasonal pattern could further obscure associations in retrospective studies lacking temporal granularity or seasonal adjustment, especially when most patients reside in regions with pronounced winter seasons [32]. For instance, depression was linked to higher MACE risk in the VDD group, likely due to increased systemic inflammation and autonomic nervous system dysfunction, which amplifies cardiovascular risks by promoting endothelial dysfunction and inflammatory feedback loops [33,34]. Glomerulonephritis, another key contributor, drives systemic inflammation and endothelial dysfunction, compounding the metabolic and vascular consequences of VDD [35,36,37]. Although other subgroups, such as those with hypertension or on ACE inhibitors and beta blockers, consistently showed non-significant trends, higher ORs in the VDD group suggest a pervasive impact of VDD on cardiovascular outcomes. The geographic homogeneity of our cohort, which was primarily US-based, may have helped reduce extreme seasonal confounding, but further research is needed to evaluate the seasonal interplay with vitamin D deficiency and MACE risk in diverse climates.

Vascular calcification remains a key contributor to cardiovascular disease in this population and is strongly associated with elevated PTH levels. High PTH levels drive vascular smooth muscle cell transformation, leading to arterial stiffness and calcification [38]. Preclinical studies show that calcimimetics reduces vascular calcification by inhibiting endothelial-to-mesenchymal transition and restoring endothelial function, although these benefits may be attenuated by VDD [39]. Clinical trials, like the ADVANCE study, emphasize the cardiovascular benefits of calcimimetics, including reductions in vascular and valve calcification; however, the pro-inflammatory and pro-calcification effects of VDD may counteract these therapeutic benefits [40].

The increased fracture incidence observed in the VDD group did not remain statistically significant after Bonferroni–Holm correction (corrected α = 0.010), despite an initial OR of 1.34 (95% CI: 1.01–1.79, *p* = 0.04). This attenuation of significance reflects the conservative nature of multiple-testing correction, which adjusts for potential type I errors across several endpoints. Given that seven outcomes were analyzed in our primary comparison, the adjusted significance threshold effectively reduced the statistical weight of marginal findings such as fracture risk.

Nonetheless, our subgroup analysis revealed that specific populations remained particularly susceptible to fractures in the context of VDD. Notably, patients with obesity (BMI > 25 kg/m^2^) had a significantly increased fracture risk (OR: 1.40, 95% CI: 1.05–1.86, *p* = 0.02); those with elevated LDL cholesterol (>100 mg/dL) also showed a higher fracture risk (OR: 1.69, 95% CI: 1.02–2.80, *p* = 0.04). These findings suggest that vitamin D deficiency may interact with underlying metabolic abnormalities to amplify skeletal fragility in specific subgroups.

Obesity contributes to compromised bone health through various mechanisms. The increased secretion of pro-inflammatory cytokines, such as TNF-α and IL-6, enhances osteoclast-mediated bone resorption; elevated leptin levels suppress osteoblast activity and impair bone formation [41,42]. Excess adiposity in the bone marrow further disrupts the bone microenvironment and impairs remodeling [43]. These effects are exacerbated by vitamin D deficiency, which impairs calcium absorption and promotes secondary hyperparathyroidism, thereby enhancing bone turnover and mineral loss.

Similarly, elevated LDL cholesterol levels contribute to skeletal fragility through oxidative stress and inflammation. Oxidized LDL directly inhibits osteoblast differentiation and mineralization while simultaneously stimulating osteoclast activity [44]. It also increases reactive oxygen species (ROS) production, which weakens bone matrix formation and leads to trabecular thinning [45,46]. In the setting of VDD, these lipid-induced effects on bone metabolism may be further magnified due to dysregulated mineral homeostasis and chronic low-grade inflammation [47].

These observations are consistent with existing studies in the literature demonstrating that dysregulated lipid metabolism and obesity impair bone integrity, particularly when compounded by vitamin D deficiency. While other subgroups, such as those with hypertension, active vitamin D use, and HbA1c > 7.0%, showed non-significant trends, the overall pattern highlights the importance of optimizing vitamin D status to mitigate bone fragility, especially in metabolically vulnerable populations. Our findings underscore the need for further investigation into targeted strategies to reduce fracture risk in high-risk dialysis patients with combined VDD, obesity, and dyslipidemia.

Although hypocalcemia was initially more prevalent in the VDD group (*p* = 0.01), it did not retain statistical significance after Bonferroni–Holm correction (corrected α = 0.0083). This suggests that VDD contributes to impaired calcium homeostasis but is not the sole factor. Additional contributors, such as dietary calcium intake, adherence to calcimimetic therapy, and baseline mineral metabolism, likely influence hypocalcemia outcomes.

No significant differences were observed in the proportion of patients achieving PTH suppression (PTH ≤ 300 pg/mL; *p* = 0.79) between groups. This finding underscores the multifactorial nature of PTH regulation in dialysis patients with SHPT, where vitamin D status alone may not be sufficient to achieve optimal suppression. Factors, such as baseline PTH levels, phosphate control, dietary calcium intake, calcimimetic adherence, and the interplay of mineral metabolism, are likely critical determinants of treatment efficacy.

Collectively, our findings demonstrate that vitamin D deficiency is not merely a biochemical abnormality but a clinically significant determinant of adverse outcomes in dialysis patients with SHPT. VDD increases mortality risk, impairs cardiovascular and bone health, and may diminish the effectiveness of calcimimetic therapy. Optimizing vitamin D status should be considered an integral component of multidimensional care in this high-risk population.

This study has several limitations. First, its reliance on observational data potentially introduces residual confounding despite the use of propensity score matching. The absence of randomization restricts the ability to infer causal relationships between vitamin D status and clinical outcomes. Second, although we categorized patients based on baseline 25(OH)D levels, we were unable to account for seasonal variations or longitudinal changes in vitamin D status due to the lack of access to individual-level or time-stamped data. Prior research suggests that such variability may attenuate observed associations through regression dilution bias. Therefore, our findings may represent conservative estimates of true associations. This limitation aligns with that of prior studies in the literature suggesting that a failure to account for intra-individual biomarker variability may underestimate true risk associations [48]. Third, variations in dietary calcium intake, calcimimetic adherence, and clinical management practices across centers were not captured and may have influenced the outcomes. Finally, the generalizability of our findings is limited, as the cohort primarily represents US-based dialysis patients with secondary hyperparathyroidism (SHPT); the results may not be extrapolated to broader or non-US chronic kidney disease populations. Future randomized controlled trials and longitudinal analyses with seasonally adjusted and repeated vitamin D measurements are warranted to validate and extend these findings.

## 5. Conclusions

This study demonstrates that VDD is significantly associated with increased all-cause mortality in dialysis patients with SHPT. While the trends of MACEs, fractures, and hypocalcemia did not retain statistical significance after correction for multiple testing, their clinical implications remain relevant, particularly among patients with obesity, dyslipidemia, and comorbidities like depression. No significant differences were observed for PTH suppression, suggesting that vitamin D status alone may not be sufficient to optimize PTH regulation. Optimizing vitamin D status, alongside calcimimetic therapy and the targeted management of modifiable risk factors, may improve clinical outcomes. Further randomized controlled trials are necessary to confirm these findings and explore effective combined interventions.

## Figures and Tables

**Table 1:** Baseline characteristics of vitamin D-deficient (VDD) and vitamin D-adequate (VDA) groups before and after propensity score matching.

	Before Matching			After Matching		
	VDD (n = 2714)	VDA (n = 2410)	Std. Diff.	VDD (n = 1744)	VDA (n = 1744)	Std. Diff.
Demographics						
Age at index date (mean ± SD)	54.7 +/− 14.1	60.0 +/− 14.3	0.37	57.2 +/− 13.4	57.3 +/− 14.3	0.01
Male, (%)	48.9%	50.5%	0.03	50.2%	50.8%	0.01
White, (%)	29.8%	42.5%	0.27	36.1%	37.1%	0.02
Black or African American, (%)	45.5%	34.2%	0.23	40.1%	39.6%	0.01
Comorbidities (%)						
Hypertension	69.6%	71.4%	0.04	71.7%	71.3%	0.01
Diabetes mellitus	38.8%	40.6%	0.04	40.7%	40.5%	<0.01
Depressive episode	11.3%	9.5%	0.06	10.7%	10.6%	<0.01
Chronic obstructive pulmonary disease	6.5%	7.1%	0.02	7.3%	7.2%	<0.01
Glomerular diseases	6.5%	5.1%	0.06	5.4%	5.9%	0.02
Systemic lupus erythematosus	3.0%	2.7%	0.02	3.1%	3.2%	<0.01
Medications (%)						
ACEI	14.1%	11.2%	0.09	12.4%	11.8%	0.02
ARB	10.9%	12.1%	0.04	11.7%	11.1%	0.02
Beta-blockers	56.7%	47.5%	0.19	50.1%	51.5%	0.03
CCB	42.5%	35.6%	0.14	39.4%	38.3%	0.02
Diuretics	9.1%	7.9%	0.04	8.7%	8.6%	<0.01
Vitamin D	29.4%	31.7%	0.05	30.3%	30.0%	<0.01
Insulin	40.6%	31.6%	0.19	35.7%	35.3%	<0.01
Cholecalciferol	11.6%	17.3%	0.16	14.7%	14.5%	<0.01
Doxercalciferol	2.1%	1.1%	0.08	1.5%	1.3%	0.02
Ergocalciferol	7.1%	8.5%	0.05	7.3%	8.1%	0.03
Calcitriol	11.2%	9.2%	0.07	10.3%	10.0%	0.01
Paricalcitol	4.5%	2.4%	0.12	3.3%	3.1%	0.01
Glucocorticoids	47.2%	40.8%	0.13	44.3%	42.8%	0.03
Laboratory, Mean ± SD						
Intact parathyroid hormone, pg/mL	567.3 +/− 668.5	384.2 +/− 443.6	0.32	508.9 +/− 587.5	404.0 +/− 466.7	0.20
Calcidiol, ng/mL	12.2 +/− 4.4	45.3 +/− 14.2	3.15	12.6 +/− 4.3	44.7 +/− 14.3	3.04
Sodium, mmol/L	137.0 +/− 4.5	137.5 +/− 3.9	0.13	137.0 +/− 4.4	137.4 +/− 3.9	0.09
Potassium, mmol/L	4.4 +/− 0.7	4.4 +/− 0.7	0.01	4.4 +/− 0.7	4.5 +/− 0.7	0.07
Calcium, mg/dL	9.3 +/− 1.3	9.5 +/− 1.2	0.17	9.3 +/− 1.3	9.5 +/− 1.2	0.09
Phosphate, mg/dL	4.6 +/− 2.2	4.2 +/− 1.8	0.21	4.5 +/− 2.1	4.3 +/− 1.9	0.12
HbA1c, %	6.4 +/− 1.9	6.2 +/− 1.5	0.11	6.4 +/− 2.0	6.1 +/− 1.6	0.13
Albumin, g/dL	3.5 +/− 0.8	3.7 +/− 0.7	0.28	3.5 +/− 0.7	3.6 +/− 0.7	0.25
LDL, mg/dL	77.2 +/− 39.7	74.2 +/− 35.4	0.08	76.3 +/− 41.2	74.9 +/− 36.8	0.04
Hemoglobin, g/dL	10.2 +/− 2.2	10.8 +/− 2.2	0.24	10.4 +/− 2.2	10.7 +/− 2.2	0.13
BMI, kg/m^2^	29.2 +/− 7.8	28.7 +/− 7.2	0.07	29.3 +/− 7.7	28.8 +/− 7.3	0.07

ACEI—angiotensin-converting enzyme inhibitor; ARB—angiotensin II receptor blocker; BMI—body mass index; CCB—calcium channel blocker; HbA1c—hemoglobin A1c; LDL—low-density lipoprotein; SD—standard deviation; Std. Diff.—standardized difference.

**Table 2:** Geographic distribution of patients in the vitamin D-deficient group.

Region	Number of Patients in VDD Group
United States (US Network)	2192 patients
Europe and Middle East (EMEA Network)	106 patients
Asia–Pacific (APAC Network)	0 patients
Latin America (LATAM Network)	0 patients
Global Total	2298 patients

## Data Availability

The data presented in this study are available on request from the corresponding author due to privacy and ethical restrictions. The dataset was obtained from the TriNetX Global Federated Health Research Network, which collects deidentified electronic medical records from multiple healthcare institutions. Access to the dataset is restricted by institutional policies and data-sharing agreements. Researchers interested in accessing the data may request the data from TriNetX, subject to institutional approval and compliance with data privacy regulations.

## Supplementary Materials

The following supporting information can be downloaded at: https://www.mdpi.com/article/10.3390/nu17091536/s1.

## Abbreviations

The following abbreviations are used in this manuscript:

Abbreviation	Full Form
ACEI	angiotensin-converting enzyme inhibitor
ARB	angiotensin II receptor blocker
CaSRs	calcium-sensing receptors
CKD	chronic kidney disease
DM	diabetes mellitus
MACEs	major adverse cardiovascular events
PTH	parathyroid hormone
SHPT	secondary hyperparathyroidism
VDD	vitamin D deficiency
VDA	vitamin D adequate
