# Supplementary material for: Vitamin D Deficiency and the Clinical Outcomes of Calcimimetic Therapy in Dialysis Patients: A Population-Based Study
Source: Nutrients. 2025 Apr 30;17(9):1536. doi: 10.3390/nu17091536 (PMC12073363; doi:10.3390/nu17091536)
Supplement: Supplementary file 1 [file nutrients-17-01536-s001.zip › supplementary Data S2 - inclusion and exclusion criteria.pdf]

Supplement S2. ICD-10-CM codes for clinical outcomes.

Clinical outcomes	ICD-10-CM-codes
All-cause mortality	Deceased (variable codified by TriNetX)<br>R99 ill-defined and unknown cause of mortality
MACE	I21 Acute myocardial infarction<br>I46 Cardiac arrest<br>I49 Other cardiac arrhythmias<br>I50 Heart failure<br>I63 Cerebral infarction<br>I61 Nontraumatic intracerebral hemorrhage
Fracture	S22 Fracture of rib(s), sternum and thoracic spine<br>S32 Fracture of lumbar spine and pelvis<br>S42 Fracture of shoulder and upper arm<br>S52 Fracture of forearm<br>S62 Fracture at wrist and hand level<br>S72 Fracture of femur<br>S82 Fracture of lower leg, including ankle<br>S92 Fracture of foot and toe, except ankle
In the study cohorts, dialysis patients were identified using ICD-10 codes. Patients with any recorded outcome of interest occurring prior to the defined time window were excluded to ensure accurate assessment of incident outcomes.	
