# Supplementary material for: Vitamin D Deficiency and the Clinical Outcomes of Calcimimetic Therapy in Dialysis Patients: A Population-Based Study
Source: Nutrients. 2025 Apr 30;17(9):1536. doi: 10.3390/nu17091536 (PMC12073363; doi:10.3390/nu17091536)
Supplement: Supplementary file 1 [file nutrients-17-01536-s001.zip › supplementary Data S3 - propensity score matching.pdf]

Cohort 1 (N = 2,714) and cohort 2 (N = 2,410) characteristics before propensity score matching							
**Demographics**							
Cohort			Mean ± SD	Patients	% of Cohort	P-Value	Std diff.
1<br>2	AI	Age at Index	54.7 +/- 14.1<br>60.0 +/- 14.3	2,714<br>2,410	100%<br>100%	<0.001	0.368
1<br>2	2106-3	White		809<br>1,024	29.8%<br>42.5%	<0.001	0.266
1<br>2	F	Female		1,366<br>1,170	50.3%<br>48.5%	0.202	0.036
1<br>2	2054-5	Black or African American		1,235<br>825	45.5%<br>34.2%	<0.001	0.232
1<br>2	M	Male		1,328<br>1,217	48.9%<br>50.5%	0.263	0.031
1<br>2	2028-9	Asian		111<br>150	4.1%<br>6.2%	0.001	0.097
**Diagnosis**							
Cohort			Mean ± SD	Patients	% of Cohort	P-Value	Std diff.
1<br>2	E08-E13	Diabetes mellitus		1,054<br>979	38.8%<br>40.6%	0.192	0.037
1<br>2	I10-I1A	Hypertensive diseases		1,889<br>1,720	69.6%<br>71.4%	0.166	0.039
1<br>2	I10-I15	Hypertensive diseases (deprecated 2018)		1,884<br>1,717	69.4%<br>71.2%	0.153	0.040
1<br>2	E03	Other hypothyroidism		243<br>254	9.0%<br>10.5%	0.056	0.053
1<br>2	J44	Other chronic obstructive pulmonary disease		176<br>171	6.5%<br>7.1%	0.385	0.024
1<br>2	F32	Depressive episode		308<br>230	11.3%<br>9.5%	0.035	0.059
1<br>2	N00-N08	Glomerular diseases		176<br>124	6.5%<br>5.1%	0.041	0.057
1<br>2	C64-C68	Malignant neoplasms of urinary tract		29<br>39	1.1%<br>1.6%	0.086	0.048
1<br>2	M32	Systemic lupus erythematosus (SLE)		81<br>65	3.0%<br>2.7%	0.537	0.017
**Medication**							
Cohort			Mean ± SD	Patients	% of Cohort	P-Value	Std diff.
1<br>2	CV100	BETA BLOCKERS/RELATED		1,540<br>1,145	56.7%<br>47.5%	<0.001	0.186
1<br>2	CV300	ANTIARRHYTHMICS		1,304<br>991	48.0%<br>41.1%	<0.001	0.140
1<br>2	CV200	CALCIUM CHANNEL BLOCKERS		1,154<br>859	42.5%<br>35.6%	<0.001	0.141
1<br>2	CV805	ANGIOTENSIN II INHIBITOR		295<br>292	10.9%<br>12.1%	0.162	0.039
1<br>2	CN104	NON-STEROIDAL ANTI-INFLAMMATORY ANALGESICS		69<br>40	2.5%<br>1.7%	0.029	0.062
1<br>2	CN302	BENZODIAZEPINE DERIVATIVE SEDATIVES/HYPNOTICS		1,098<br>757	40.5%<br>31.4%	<0.001	0.189
1<br>2	HS501	INSULIN		1,103<br>762	40.6%<br>31.6%	<0.001	0.189

1<br>2	4821	glipizide		44<br>55	1.6%<br>2.3%	0.086	0.048
1<br>2	593411	sitagliptin		36<br>32	1.3%<br>1.3%	0.997	<0.001
1<br>2	1100699	linagliptin		22<br>23	0.8%<br>1.0%	0.582	0.015
1<br>2	1545653	empagliflozin		10<br>11	0.4%<br>0.5%	0.623	0.014
1<br>2	VT500	VITAMIN D		797<br>765	29.4%<br>31.7%	0.065	0.052
1<br>2	2418	cholecalciferol		315<br>417	11.6%<br>17.3%	<0.001	0.163
1<br>2	11516	doxercalciferol		58<br>27	2.1%<br>1.1%	0.004	0.080
1<br>2	VT504	ERGOCALCIFEROL		193<br>205	7.1%<br>8.5%	0.063	0.052
1<br>2	VT502	CALCITRIOL		303<br>221	11.2%<br>9.2%	0.019	0.066
1<br>2	73710	paricalcitol		123<br>58	4.5%<br>2.4%	<0.001	0.116
1<br>2	HS051	GLUCOCORTICOIDS		1,282<br>984	47.2%<br>40.8%	<0.001	0.129
1<br>2	CV709	DIURETICS, OTHER		246<br>190	9.1%<br>7.9%	0.131	0.042
1<br>2	CV800	ACE INHIBITORS		384<br>269	14.1%<br>11.2%	0.001	0.090
1<br>2	CN103	NON-OPIOID ANALGESICS		1,925<br>1,325	70.9%<br>55.0%	<0.001	0.335
1<br>2	BL117	PLATELET AGGREGATION INHIBITORS		1,044<br>712	38.5%<br>29.5%	<0.001	0.189
**Laboratory**							
Cohort			Mean ± SD	Patients	% of Cohort	P-Value	Std diff.
1<br>2	9039	Parathyrin intact [Mass/volume] in Serum or Plasma	567.3 +/- 668.5<br>384.2 +/- 443.6	1,046<br>791	38.5%<br>32.8%	<0.001	0.323
1<br>2		0 - 0 pg/mL		1,047<br>791	38.6%<br>32.8%	<0.001	0.120
1<br>2	9034	Calcidiol [Mass/volume] in Serum or Plasma	12.2 +/- 4.4<br>45.3 +/- 14.2	405<br>417	14.9%<br>17.3%	<0.001	3.151
1<br>2		0 - 0 ng/mL		454<br>418	16.7%<br>17.3%	0.558	0.016
1<br>2	9029	Sodium [Moles/volume] in Serum, Plasma or Blood	137.0 +/- 4.5<br>137.5 +/- 3.9	2,293<br>1,816	84.5%<br>75.4%	<0.001	0.130
1<br>2		0 - 0 mmol/L		2,301<br>1,816	84.8%<br>75.4%	<0.001	0.238
1<br>2	9028	Potassium [Moles/volume] in Serum, Plasma or Blood	4.4 +/- 0.7<br>4.4 +/- 0.7	2,349<br>1,835	86.6%<br>76.1%	0.728	0.011
1<br>2		0 - 0 mmol/L		2,349<br>1,835	86.6%<br>76.1%	<0.001	0.270
1<br>2	9023	Chloride [Moles/volume] in Serum, Plasma or Blood	99.1 +/- 9.8<br>100.5 +/- 6.2	2,339<br>1,821	86.2%<br>75.6%	<0.001	0.175
1<br>2		0 - 0 mmol/L		2,339<br>1,821	86.2%<br>75.6%	<0.001	0.273

1<br>2	9021	Bicarbonate [Moles/volume] in Serum, Plasma or Blood	24.1 +/- 4.3<br>24.5 +/- 4.0	2,252<br>1,811	83.0%<br>75.1%	0.002	0.101
1<br>2		0 - 0 mmol/L		2,252<br>1,811	83.0%<br>75.1%	<0.001	0.193
1<br>2	9030	Urea nitrogen [Mass/volume] in Serum, Plasma or Blood	42.5 +/- 25.6<br>41.0 +/- 25.4	2,222<br>1,798	81.9%<br>74.6%	0.076	0.056
1<br>2		0 - 0 mg/dL		2,222<br>1,798	81.9%<br>74.6%	<0.001	0.177
1<br>2	9024	Creatinine [Mass/volume] in Serum, Plasma or Blood	6.6 +/- 9.6<br>4.9 +/- 3.9	2,252<br>1,759	83.0%<br>73.0%	<0.001	0.232
1<br>2		0 - 0 mg/dL		2,252<br>1,759	83.0%<br>73.0%	<0.001	0.243
1<br>2	9025	Glucose [Mass/volume] in Serum, Plasma or Blood	115.6 +/- 58.1<br>116.8 +/- 48.4	2,352<br>1,826	86.7%<br>75.8%	0.465	0.023
1<br>2		0 - 0 mg/dL		2,352<br>1,827	86.7%<br>75.8%	<0.001	0.281
1<br>2	9027	Phosphate [Mass/volume] in Serum, Plasma or Blood	4.6 +/- 2.2<br>4.2 +/- 1.8	1,955<br>1,457	72.0%<br>60.5%	<0.001	0.205
1<br>2		0 - 0 mg/dL		1,955<br>1,457	72.0%<br>60.5%	<0.001	0.247
1<br>2	9022	Calcium [Mass/volume] in Serum, Plasma or Blood	9.3 +/- 1.3<br>9.5 +/- 1.2	2,324<br>1,815	85.6%<br>75.3%	<0.001	0.173
1<br>2		0 - 0 mg/dL		2,335<br>1,815	86.0%<br>75.3%	<0.001	0.274
1<br>2	9012	Erythrocytes [#/volume] in Blood	3.4 +/- 0.9<br>3.6 +/- 0.9	2,280<br>1,774	84.0%<br>73.6%	<0.001	0.156
1<br>2		0 - 0 10*6/uL		2,282<br>1,774	84.1%<br>73.6%	<0.001	0.259
1<br>2	9014	Hemoglobin [Mass/volume] in Blood	10.2 +/- 2.2<br>10.8 +/- 2.2	2,228<br>1,723	82.1%<br>71.5%	<0.001	0.243
1<br>2		0 - 0 g/dL		2,228<br>1,723	82.1%<br>71.5%	<0.001	0.253
1<br>2	9020	Platelets [#/volume] in Blood	212.9 +/- 95.0<br>212.2 +/- 86.7	2,278<br>1,772	83.9%<br>73.5%	0.822	0.007
1<br>2		0 - 0 10*3/uL		2,281<br>1,775	84.0%<br>73.7%	<0.001	0.257
1<br>2	9044	Alanine aminotransferase [Enzymatic activity/volume] in Serum, Plasma or Blood	23.7 +/- 42.5<br>24.1 +/- 63.6	1,929<br>1,472	71.1%<br>61.1%	0.820	0.008
1<br>2		0 - 0 U/L		1,929<br>1,472	71.1%<br>61.1%	<0.001	0.212
1<br>2	9047	Aspartate aminotransferase [Enzymatic activity/volume] in Serum or Plasma	28.0 +/- 48.3<br>27.1 +/- 70.2	1,981<br>1,489	73.0%<br>61.8%	0.662	0.015
1<br>2		0 - 0 U/L		1,981<br>1,489	73.0%<br>61.8%	<0.001	0.241
1<br>2	9046	Alkaline phosphatase [Enzymatic	156.5 +/- 156.6<br>128.3 +/- 101.4	1,987<br>1,503	73.2%<br>62.4%	<0.001	0.214

		activity/volume] in Serum, Plasma or Blood					
1<br>2		0 - 0 U/L		1,987<br>1,503	73.2%<br>62.4%	<0.001	0.234
1<br>2	9050	Bilirubin total [Mass/volume] in Serum, Plasma or Blood	0.7 +/- 2.4<br>0.6 +/- 0.5	1,922<br>1,437	70.8%<br>59.6%	0.054	0.071
1<br>2		0 - 0 mg/dL		1,930<br>1,437	71.1%<br>59.6%	<0.001	0.243
1<br>2	9045	Albumin [Mass/volume] in Serum, Plasma or Blood	3.5 +/- 0.8<br>3.7 +/- 0.7	2,019<br>1,620	74.4%<br>67.2%	<0.001	0.277
1<br>2		0 - 0 g/dL		2,020<br>1,620	74.4%<br>67.2%	<0.001	0.159
1<br>2	9033	Prothrombin time (PT) in Plasma or Blood	14.7 +/- 6.1<br>14.9 +/- 6.3	1,513<br>980	55.7%<br>40.7%	0.452	0.031
1<br>2		0 - 0 s		1,513<br>981	55.7%<br>40.7%	<0.001	0.305
1<br>2	9000	Cholesterol [Mass/volume] in Serum or Plasma	147.8 +/- 53.2<br>146.9 +/- 47.6	707<br>536	26.1%<br>22.2%	0.751	0.018
1<br>2		0 - 0 mg/dL		707<br>536	26.1%<br>22.2%	0.001	0.089
1<br>2	9002	Cholesterol in LDL [Mass/volume] in Serum or Plasma	77.2 +/- 39.7<br>74.2 +/- 35.4	636<br>501	23.4%<br>20.8%	0.193	0.078
1<br>2		0 - 0 mg/dL		637<br>501	23.5%<br>20.8%	0.021	0.065
1<br>2	9037	Hemoglobin A1c/Hemoglobin total in Blood	6.4 +/- 1.9<br>6.2 +/- 1.5	901<br>697	33.2%<br>28.9%	0.038	0.107
1<br>2		0 - 0 %		901<br>697	33.2%<br>28.9%	0.001	0.093
1<br>2	2003	Left Ventricular Ejection Fraction (LVEF) (%)	51.0 +/- 16.1<br>54.4 +/- 16.4	131<br>87	4.8%<br>3.6%	0.125	0.213
1<br>2		0 - 0 %		131<br>87	4.8%<br>3.6%	0.031	0.061
1<br>2	9083	BMI	29.2 +/- 7.8<br>28.7 +/- 7.2	1,777<br>1,493	65.5%<br>62.0%	0.055	0.068
1<br>2		0 - 0 kg/m2		1,778<br>1,493	65.5%<br>62.0%	0.008	0.074
1<br>2	8001	Glomerular filtration rate/1.73 sq M predicted [Volume Rate/Area] in Serum, Plasma or Blood by Creatinine-based formula (MDRD)	21.2 +/- 25.0<br>25.6 +/- 26.3	2,269<br>1,768	83.6%<br>73.4%	<0.001	0.173
1<br>2		0 - 0 mL/min/{1.73_m2}		2,269<br>1,768	83.6%<br>73.4%	<0.001	0.251
1<br>2	2001	Corrected QT Interval (QTc)	465.0 +/- 40.3<br>459.3 +/- 41.3	392<br>282	14.4%<br>11.7%	0.071	0.141
1<br>2		0 - 0 ms		392<br>282	14.4%<br>11.7%	0.004	0.081
1<br>2	5195-3	Hepatitis B virus surface Ag [Presence] in Serum	-0.0 +/- 0.3<br>0 +/- 0.2	172<br>220	6.3%<br>9.1%	0.645	0.046
1<br>2		0 - 0 null		172<br>220	6.3%<br>9.1%	<0.001	0.105

1<br>2	16128-1	Hepatitis C virus Ab [Presence] in Serum	-0.1 +/- 0.5<br>-0.2 +/- 0.5	147<br>130	5.4%<br>5.4%	0.138	0.179
1<br>2		0 - 0 null		152<br>135	5.6%<br>5.6%	0.999	<0.001

Cohort 1 (N = 1,744) and cohort 2 (N = 1,744) characteristics after propensity score matching

Demographics

Cohort			Mean ± SD	Patients	% of Cohort	P-Value	Std diff.
1<br>2	AI	Age at Index	57.2 +/- 13.4<br>57.3 +/- 14.3	1,744<br>1,744	100%<br>100%	0.775	0.010
1<br>2	2106-3	White		630<br>647	36.1%<br>37.1%	0.550	0.020
1<br>2	F	Female		855<br>844	49.0%<br>48.4%	0.709	0.013
1<br>2	2054-5	Black or African American		699<br>691	40.1%<br>39.6%	0.782	0.009
1<br>2	M	Male		875<br>886	50.2%<br>50.8%	0.710	0.013
1<br>2	2028-9	Asian		89<br>94	5.1%<br>5.4%	0.704	0.013

Diagnosis

Cohort			Mean ± SD	Patients	% of Cohort	P-Value	Std diff.
1<br>2	E08-E13	Diabetes mellitus		710<br>707	40.7%<br>40.5%	0.918	0.004
1<br>2	I10-I1A	Hypertensive diseases		1,250<br>1,244	71.7%<br>71.3%	0.822	0.008
1<br>2	I10-I15	Hypertensive diseases (deprecated 2018)		1,248<br>1,242	71.6%<br>71.2%	0.822	0.008
1<br>2	E03	Other hypothyroidism		172<br>174	9.9%<br>10.0%	0.910	0.004
1<br>2	J44	Other chronic obstructive pulmonary disease		128<br>125	7.3%<br>7.2%	0.845	0.007
1<br>2	F32	Depressive episode		187<br>185	10.7%<br>10.6%	0.913	0.004
1<br>2	N00-N08	Glomerular diseases		94<br>103	5.4%<br>5.9%	0.509	0.022
1<br>2	C64-C68	Malignant neoplasms of urinary tract		25<br>21	1.4%<br>1.2%	0.553	0.020
1<br>2	M32	Systemic lupus erythematosus (SLE)		54<br>55	3.1%<br>3.2%	0.922	0.003

Medication

Cohort			Mean ± SD	Patients	% of Cohort	P-Value	Std diff.
1<br>2	CV100	BETA BLOCKERS/RELATED		873<br>898	50.1%<br>51.5%	0.397	0.029
1<br>2	CV300	ANTIARRHYTHMICS		773<br>778	44.3%<br>44.6%	0.865	0.006
1<br>2	CV200	CALCIUM CHANNEL BLOCKERS		688<br>668	39.4%<br>38.3%	0.487	0.024
1<br>2	CV805	ANGIOTENSIN II INHIBITOR		204<br>193	11.7%<br>11.1%	0.558	0.020
1<br>2	CN104	NON-STEROIDAL ANTI-INFLAMMATORY ANALGESICS		30<br>35	1.7%<br>2.0%	0.531	0.021

1<br>2	CN302	BENZODIAZEPINE DERIVATIVE SEDATIVES/HYPNOTICS		618<br>625	35.4%<br>35.8%	0.805	0.008
1<br>2	HS501	INSULIN		622<br>616	35.7%<br>35.3%	0.832	0.007
1<br>2	4821	glipizide		31<br>35	1.8%<br>2.0%	0.619	0.017
1<br>2	593411	sitagliptin		19<br>22	1.1%<br>1.3%	0.637	0.016
1<br>2	1100699	linagliptin		17<br>14	1.0%<br>0.8%	0.588	0.018
1<br>2	1545653	empagliflozin		10<br>10	0.6%<br>0.6%	1	<0.001
1<br>2	VT500	VITAMIN D		529<br>523	30.3%<br>30.0%	0.825	0.007
1<br>2	2418	cholecalciferol		256<br>253	14.7%<br>14.5%	0.886	0.005
1<br>2	11516	doxercalciferol		26<br>23	1.5%<br>1.3%	0.666	0.015
1<br>2	VT504	ERGOCALCIFEROL		127<br>141	7.3%<br>8.1%	0.373	0.030
1<br>2	VT502	CALCITRIOL		180<br>174	10.3%<br>10.0%	0.737	0.011
1<br>2	73710	paricalcitol		57<br>54	3.3%<br>3.1%	0.772	0.010
1<br>2	HS051	GLUCOCORTICOIDS		773<br>746	44.3%<br>42.8%	0.357	0.031
1<br>2	CV709	DIURETICS,OTHER		152<br>150	8.7%<br>8.6%	0.904	0.004
1<br>2	CV800	ACE INHIBITORS		217<br>205	12.4%<br>11.8%	0.533	0.021
1<br>2	CN103	NON-OPIOID ANALGESICS		1,084<br>1,096	62.2%<br>62.8%	0.675	0.014
1<br>2	BL117	PLATELET AGGREGATION INHIBITORS		580<br>575	33.3%<br>33.0%	0.857	0.006

Laboratory

Cohort			Mean ± SD	Patients	% of Cohort	P-Value	Std diff.
1<br>2	9039	Parathyrin.intact [Mass/volume] in Serum or Plasma	508.9 +/- 587.5<br>404.0 +/- 466.7	606<br>610	34.7%<br>35.0%	0.001	0.198
1<br>2		0 - 0 pg/mL		606<br>610	34.7%<br>35.0%	0.887	0.005
1<br>2	9034	Calcidiol [Mass/volume] in Serum or Plasma	12.6 +/- 4.3<br>44.7 +/- 14.3	237<br>312	13.6%<br>17.9%	<0.001	3.040
1<br>2		0 - 0 ng/mL		266<br>313	15.3%<br>17.9%	0.032	0.072
1<br>2	9029	Sodium [Moles/volume] in Serum, Plasma or Blood	137.0 +/- 4.4<br>137.4 +/- 3.9	1,406<br>1,392	80.6%<br>79.8%	0.024	0.086
1<br>2		0 - 0 mmol/L		1,408<br>1,392	80.7%<br>79.8%	0.496	0.023
1<br>2	9028	Potassium [Moles/volume] in Serum, Plasma or Blood	4.4 +/- 0.7<br>4.5 +/- 0.7	1,421<br>1,408	81.5%<br>80.7%	0.057	0.071
1<br>2		0 - 0 mmol/L		1,421<br>1,408	81.5%<br>80.7%	0.574	0.019

1<br>2	9023	Chloride [Moles/volume] in Serum, Plasma or Blood	99.6 +/- 8.2<br>100.1 +/- 6.3	1,412<br>1,397	81.0%<br>80.1%	0.070	0.068
1<br>2		0 - 0 mmol/L		1,412<br>1,397	81.0%<br>80.1%	0.521	0.022
1<br>2	9021	Bicarbonate [Moles/volume] in Serum, Plasma or Blood	24.1 +/- 4.2<br>24.6 +/- 4.0	1,404<br>1,387	80.5%<br>79.5%	0.005	0.107
1<br>2		0 - 0 mmol/L		1,404<br>1,387	80.5%<br>79.5%	0.472	0.024
1<br>2	9030	Urea nitrogen [Mass/volume] in Serum, Plasma or Blood	42.3 +/- 25.4<br>41.5 +/- 25.4	1,397<br>1,378	80.1%<br>79.0%	0.385	0.033
1<br>2		0 - 0 mg/dL		1,397<br>1,378	80.1%<br>79.0%	0.425	0.027
1<br>2	9024	Creatinine [Mass/volume] in Serum, Plasma or Blood	6.0 +/- 7.4<br>5.2 +/- 4.0	1,360<br>1,348	78.0%<br>77.3%	0.002	0.122
1<br>2		0 - 0 mg/dL		1,360<br>1,348	78.0%<br>77.3%	0.626	0.017
1<br>2	9025	Glucose [Mass/volume] in Serum, Plasma or Blood	116.8 +/- 54.1<br>116.3 +/- 48.1	1,416<br>1,407	81.2%<br>80.7%	0.795	0.010
1<br>2		0 - 0 mg/dL		1,416<br>1,408	81.2%<br>80.7%	0.730	0.012
1<br>2	9027	Phosphate [Mass/volume] in Serum, Plasma or Blood	4.5 +/- 2.1<br>4.3 +/- 1.9	1,144<br>1,136	65.6%<br>65.1%	0.006	0.115
1<br>2		0 - 0 mg/dL		1,144<br>1,136	65.6%<br>65.1%	0.776	0.010
1<br>2	9022	Calcium [Mass/volume] in Serum, Plasma or Blood	9.3 +/- 1.3<br>9.5 +/- 1.2	1,404<br>1,394	80.5%<br>79.9%	0.020	0.088
1<br>2		0 - 0 mg/dL		1,407<br>1,394	80.7%<br>79.9%	0.580	0.019
1<br>2	9012	Erythrocytes [#/volume] in Blood	3.5 +/- 0.9<br>3.6 +/- 0.9	1,378<br>1,371	79.0%<br>78.6%	0.001	0.125
1<br>2		0 - 0 10*6/uL		1,378<br>1,371	79.0%<br>78.6%	0.772	0.010
1<br>2	9014	Hemoglobin [Mass/volume] in Blood	10.4 +/- 2.2<br>10.7 +/- 2.2	1,337<br>1,325	76.7%<br>76.0%	0.001	0.134
1<br>2		0 - 0 g/dL		1,337<br>1,325	76.7%<br>76.0%	0.633	0.016
1<br>2	9020	Platelets [#/volume] in Blood	211.5 +/- 90.9<br>212.5 +/- 87.4	1,375<br>1,366	78.8%<br>78.3%	0.768	0.011
1<br>2		0 - 0 10*3/uL		1,378<br>1,369	79.0%<br>78.5%	0.709	0.013
1<br>2	9044	Alanine aminotransferase [Enzymatic activity/volume] in Serum, Plasma or Blood	23.7 +/- 39.0<br>25.1 +/- 71.5	1,151<br>1,147	66.0%<br>65.8%	0.542	0.025
1<br>2		0 - 0 U/L		1,151<br>1,147	66.0%<br>65.8%	0.886	0.005
1<br>2	9047	Aspartate aminotransferase [Enzymatic activity/volume] in Serum or Plasma	28.1 +/- 41.9<br>27.8 +/- 78.5	1,171<br>1,168	67.1%<br>67.0%	0.896	0.005

1<br>2		0 - 0 U/L		1,171<br>1,168	67.1%<br>67.0%	0.914	0.004
1<br>2	9046	Alkaline phosphatase [Enzymatic activity/volume] in Serum, Plasma or Blood	153.3 +/- 149.1<br>132.3 +/- 107.6	1,184<br>1,182	67.9%<br>67.8%	<0.001	0.162
1<br>2		0 - 0 U/L		1,184<br>1,182	67.9%<br>67.8%	0.942	0.002
1<br>2	9050	Bilirubin.total [Mass/volume] in Serum, Plasma or Blood	0.7 +/- 1.9<br>0.6 +/- 0.5	1,135<br>1,139	65.1%<br>65.3%	0.036	0.088
1<br>2		0 - 0 mg/dL		1,136<br>1,139	65.1%<br>65.3%	0.915	0.004
1<br>2	9045	Albumin [Mass/volume] in Serum, Plasma or Blood	3.5 +/- 0.7<br>3.6 +/- 0.7	1,241<br>1,234	71.2%<br>70.8%	<0.001	0.252
1<br>2		0 - 0 g/dL		1,242<br>1,234	71.2%<br>70.8%	0.765	0.010
1<br>2	9033	Prothrombin time (PT) in Plasma or Blood	14.8 +/- 6.0<br>14.8 +/- 5.7	809<br>824	46.4%<br>47.2%	0.817	0.011
1<br>2		0 - 0 s		809<br>825	46.4%<br>47.3%	0.587	0.018
1<br>2	9000	Cholesterol [Mass/volume] in Serum or Plasma	147.3 +/- 53.6<br>148.1 +/- 48.2	409<br>406	23.5%<br>23.3%	0.824	0.016
1<br>2		0 - 0 mg/dL		409<br>406	23.5%<br>23.3%	0.904	0.004
1<br>2	9002	Cholesterol in LDL [Mass/volume] in Serum or Plasma	76.3 +/- 41.2<br>74.9 +/- 36.8	381<br>376	21.8%<br>21.6%	0.624	0.036
1<br>2		0 - 0 mg/dL		381<br>376	21.8%<br>21.6%	0.837	0.007
1<br>2	9037	Hemoglobin A1c/Hemoglobin total in Blood	6.4 +/- 2.0<br>6.1 +/- 1.6	547<br>539	31.4%<br>30.9%	0.031	0.131
1<br>2		0 - 0 %		547<br>539	31.4%<br>30.9%	0.770	0.010
1<br>2	2003	Left Ventricular Ejection Fraction (LVEF) (%)	53.4 +/- 15.0<br>54.6 +/- 16.2	69<br>69	4.0%<br>4.0%	0.661	0.075
1<br>2		0 - 0 %		69<br>69	4.0%<br>4.0%	1	<0.001
1<br>2	9083	BMI	29.3 +/- 7.7<br>28.8 +/- 7.3	1,104<br>1,118	63.3%<br>64.1%	0.106	0.069
1<br>2		0 - 0 kg/m2		1,104<br>1,118	63.3%<br>64.1%	0.622	0.017
1<br>2	8001	Glomerular filtration rate/1.73 sq M predicted [Volume Rate/Area] in Serum, Plasma or Blood by Creatinine-based formula (MDRD)	22.5 +/- 25.5<br>24.4 +/- 26.5	1,371<br>1,358	78.6%<br>77.9%	0.052	0.074
1<br>2		0 - 0 mL/min/{1.73_m2}		1,371<br>1,358	78.6%<br>77.9%	0.594	0.018
1<br>2	2001	Corrected QT Interval (QTc)	462.8 +/- 42.0<br>459.1 +/- 39.2	222<br>220	12.7%<br>12.6%	0.336	0.092
1<br>2		0 - 0 ms		222<br>220	12.7%<br>12.6%	0.919	0.003

1<br>2	5195-3	Hepatitis B virus surface Ag [Presence] in Serum	-0.0 +/- 0.3<br>0.0 +/- 0.2	141<br>148	8.1%<br>8.5%	0.224	0.143
1<br>2		0 - 0 null		141<br>148	8.1%<br>8.5%	0.667	0.015
1<br>2	16128-1	Hepatitis C virus Ab [Presence] in Serum	-0.2 +/- 0.5<br>-0.2 +/- 0.5	104<br>108	6.0%<br>6.2%	0.919	0.014
1<br>2		0 - 0 null		109<br>113	6.3%<br>6.5%	0.781	0.009
